# How will COVID-19 affect an already fragile global economy?

**DOI:** 10.1007/s10368-021-00509-2

**Published:** 2021-09-28

**Authors:** Joaquim Oliveira Martins, Werner Roeger

**Affiliations:** 1 CEPII and University Paris-Dauphine-PSL, Paris, France; 2 DIW, EIIW, VIVES-KU Leuven, Leuven, Belgium

The COVID-19 pandemic and associated lock down measures have hit the global economy and affected the core of our socio economic and business models, which were already exposed to a number of challenging developments. Namely, industrialised countries are experiencing a secular decline of interest and growth rates, possibly related to medium term demographic and technology developments, rising demand for save assets and increasing divergence among people, firms and regions. With the demographic transition, pension and health care are putting additional strain on government budgets. These trends are increasingly restricting monetary and fiscal policies to stabilise the economy in the case of adverse shocks. On the technology side, digitalisation of consumption and production requires structural adjustments on various fronts in order to remain competitive and generate new opportunities for displaced jobs. Since a number of years, globalisation is experiencing sizeable headwinds with rising protectionism and increasing scepticism of universal benefits related to ever increasing international trade and financial integration. Focusing on the interactions between these trends and the COVID-19 shock, this special issue of the journal *International Economics and Economic Policy (IEEP)* tackles mainly macroeconomic, trade and technology aspects. How will COVID-19 affect the economy given these major trends, will it be a temporary downturn or will it have more permanent effects? Will it accelerate change or hinder adjustments? All papers were presented at an on-line conference organized by the by the *IEEP* and the *EIIW* on June 11, 2021.

The special issue starts with a paper by *Karimov and Konings* analysing the profile of the employment shock, using a detailed Belgian dataset which allows to distinguish between start-ups and incumbent firms (grouped by age classes). The paper finds that the employment losses are mainly located with incumbent firms but predicts a more structural, medium term negative effect associated missing generation of start-ups. This provides a more nuanced assessment of the shape of the recovery in particular it contrasts with US evidence on business creation in 2020.

The next set of papers discusses the constraints which complicate the policy response and the impact this has on the shape of the recession. The paper by *Kollmann* identifies the nature of the shock through the lens of a new Keynesian macroeconomic model where policy is constrained by an (expectations driven) liquidity trap. The paper finds that the observed output and inflation responses are most consistent with a persistent negative supply shock giving additional support to the view that negative effects might be longer lasting.

*Bismut and Ramajo* analyse how COVID-19 might affect the future evolution of real interest rates. Based on an econometric analysis of interest rates for 19 OECD countries over the period 1970–2017 they identify major drivers of interest rates, namely potential output, risk effects and budget deficits. Their estimated equation captures well past developments and predicts that under a scenario of continued low productivity it will be unlikely that the current level of interest rates will change significantly. However pressures from deficits and public debt could emerge, but their effect will depend on saving behaviour.

*Biase and Doughtery* introduces a more granular dimension of the fiscal adjustment by considering the constraints and behaviour of subnational governments. Indeed, subnational entities have more limited fiscal space than central governments. Given social expenditure pressures associated with COVID-19 as well as reduced fiscal revenues at all levels, subnational governments are often forced to reduce public investment, thus making fiscal policy design more pro-cyclical than at the central government level. This is why most of the COVID-19 related fiscal spending has been undertaken by central governments.

*Van Ark, de Vries, and Erumban* compare productivity developments between the US, France and the UK using detailed industry data. The paper finds positive productivity effects of the COVID-19 shock resulting from reduced production of low productivity sectors (hospitality, culture and other services). However, only the US shows positive within industry effects and the US and the UK show stronger effects for digitally intensive industries. This could be related to more favourable institutional and policy settings.

*Doehring, Hristov, Maier, Roeger and Thum-Thysen* use a two sector model distinguishing between a conventional and a digital service sector. The paper emphasises structural differences concerning scalability, entry barriers and skill demands across these two sectors and explores the economic consequences of a permanent shift towards digital (consumer) services. The paper shows how the distribution of economic rents between workers with and without digital skills and platforms are determined by labour supply conditions and entry barriers.

Finally, *de Braga de Macedo, Oliveira Martins and Tovar Jalles* provide a global and long term view on the interactions among globalisation, economic convergence and freedoms. They find that, over the past decades, globalisation, freedoms and economic convergence have supported each other, thus creating a systemic virtuous cycle. The links between globalisation and economic convergence appear particularly strong, supporting the view that the previous and the COVID-19 induced set backs on globalisation may contribute to a continued slowdown of economic growth. The latter may have potentially negative effects on the state of political and civil liberties. Only for non OECD countries there is evidence that the relationship between the level of freedoms and economic convergence could be negative for certain stages of development.

Overall, this special issue gathers a rather rich set of insights and policy-relevant analysis. We hope it may contribute to a better understanding on how the COVID-19 shock has affected the economic outlook and may in the future interact with the ongoing major global trends that are reshaping our economies.

